# Clinical utility of remote monitoring using multiparametric implantable defibrillators algorithm

**DOI:** 10.1002/clc.23960

**Published:** 2022-12-14

**Authors:** Teruhiko Imamura

**Affiliations:** ^1^ Second Department of Internal Medicine University of Toyama Toyama Japan

To the Editor,

Methodology to monitor impending heart failure decompensation is receiving great concern to improve clinical outcomes in patients with chronic heart failure. Guerra et al.^1^ demonstrated that decongestive treatment adjustments triggered by the alerts of HeartLogic algorithm, which combines clinical data from multiple implantable defibrillator‐based sensors, was safe and effective. Of note, the early decongestive treatment and the use of high dose of diuretics were associated with more favorable outcomes. Several concerns have been raised.

One of the major concerns of this technology is its sensitivity and specificity to identify worsening heart failure. Of 242 alerts, 105 (44%) were not associated with heart failure‐related events.^1^ Could the authors explain the reasons for such a high false alarm? Some heart failure events might not be detected appropriately by this technology. Did the authors assess the negative predictive value of this technology?

Other modalities have also been introduced to monitor the degree of pulmonary congestion and adjust the dose of diuretics, including CardioMEMS and Remote Dielectric Sensing.^2^ These modalities are available irrespective of the necessity of pacemaker devices. Does HeartLogic technology have any advantages over these other modalities? Multi‐parametric algorithm of HeartLogic technology might improve the predictability of impending heart failure compared with single parameter monitoring like above modalities.

In their manuscript, high doses of diuretics are recommended to manage the alerts, whereas they might rather activate renin‐angiotensin system and worsen future cardiovascular outcomes.^3^ We can use currently a variety of anti‐heart failure agents that improve congestion without stimulating renin‐angiotensin systems, including vasopressin type 2 receptor antagonists and sodium‐glucose cotransporter 2 receptors. Optimal therapeutic strategy for the HeartLogic alerts would be the next concern.
